# Artificial Intelligence and Machine Learning: An Instructor’s Exoskeleton in the Future of Education

**DOI:** 10.1007/978-3-030-58948-6_5

**Published:** 2021-03-12

**Authors:** Stephanie E. August, Audrey Tsaima

**Affiliations:** 1 Pennsylvania State University, Altoona, PA USA; 2 Valdosta State University, Valdosta, GA USA; 3 Department of Computer Science, Loyola Marymount University and California State University, Los Angeles, CA USA; 4 BetterUp, San Francisco, CA USA

**Keywords:** Interactive learning environments, Intelligent systems applications, Learning management systems, Artificial intelligence-augmented learning, Computer-managed instruction, Interaction paradigms, Computer-assisted instruction, E-learning, Student assessment, Education, Machine learning, Intelligent agents

## Abstract

The role of artificial intelligence in US education is expanding. As education moves toward providing customized learning paths, the use of artificial intelligence (AI) and machine learning (ML) algorithms in learning systems increases. This can be viewed as growing metaphorical exoskeletons for instructors, enabling them to provide a higher level of guidance, feedback, and autonomy to learners. In turn, the instructor gains time to sense student needs and support authentic learning experiences that go beyond what AI and ML can provide. Applications of AI-based education technology support learning through automated tutoring, personalizing learning, assessing student knowledge, and automating tasks normally performed by the instructor. This technology raises questions about how it is best used, what data provides evidence of the impact of AI and ML on learning, and future directions in interactive learning systems. Exploration of the use of AI and ML for both co-curricular and independent learnings in content presentation and instruction; interactions, communications, and discussions; learner activities; assessment and evaluation; and co-curricular opportunities provide guidance for future research.

## Emerging Trends and Pedagogies

### How Do Artificial Intelligence and Machine Learning Appear in Interactive Learning Experiences?

Technology is transforming how we solve complex problems, as well as how we share information. In this chapter, we look at an innovative learning environment from the perspectives of an enrolled student, a teaching assistant, and the professor of a fluid dynamics course with 100+ enrolled students. The scenario and research provide insight into the value of incorporating artificial intelligence and machine learning into the learning experience.

- **Student**
- Halfway through the pursuit of their undergraduate degree in chemical engineering, Alex Rhimes, age 20, from Baltimore, Mary’s Lake, was planning on taking the foundational fluid dynamics class—the most notoriously difficult class in the major. The learning management system and recommendation engine used by the university suggested taking this course early based on Alex’s good grades and internship experience. Like most students, Alex logged into ratemyprofessors.com before selecting the class. With ratings in the high 4s, Alex tabbed over to the university course site and clicked the big blue “Register” button on the screen. According to the reviews, Prof. Gomez went above and beyond to create a highly personalized environment for each student. As soon as Alex registers, an email notification is received: Pre-Course Simulation Game.
- **Instructor**
- In Charrysville, Virgonne, Dr. Riley Gomez, second year associate professor, wakes up early on the first day of class to check new emails. Rolling over in bed groggily, Prof. Gomez reaches for the phone on the bedside table, scrolling past the unfiltered junk emails. Prof. Gomez is anticipating notifications from students submitting their last-minute survey responses to the self-assessment exercise shared a week ago. As student enrollments have increased and class sizes increased, Prof. Gomez started incorporating artificial intelligence and machine learning methods into the classroom environment. It was the only feasible way to reach the 100+ students enrolled in the fluid dynamics class.
- “Lecture-style classes with the sage on the stage are a thing of the past,” explains Prof. Gomez. “Using algorithms is the most effective way to manage classes in the face of increased enrollment.”
- Prof. Gomez swipes left, left, and down in order to load the most recent results. It looks like the class is spread all over the place with experience levels and interest in fluid dynamics. It is not uncommon for students to drop out of this class and fail to persist. However, Prof. Gomez is adamant about ensuring that every student feel supported and hopeful that the simulation assessment provided an opportunity for students to learn some basics before the first session. The left chart on the dashboard shows passive traits that were monitored during the simulation. Indicators such as eye movements and facial expressions are tracked in blue and orange. The middle chart on the dashboard illustrates anticipated knowledge gaps and opportunities for support based on data mined from last year’s class.

The role of artificial intelligence (AI ) in US education is continuing to expand (*Artificial Intelligence Market in the US Education Sector 2018-2022—Key Vendors are Cogni, IBM, Microsoft, Nuance Communications, Pixatel, and Quantum Adaptive Learning—ResearchAndMarkets.com* 2018). As education moves toward providing customized learning paths, the use of artificial intelligence (AI) in learning systems increases, creating scaffolding that extends the ability and reach of an instructor (Tsinakos 2006) much as a physical exoskeleton combined with augmented reality enables a worker to see more than what is in front of them, and accomplish tasks they are not able to complete on their own (Srinivasan 2018). Chatbots (Bradeško and Mladenić 2012; Fonte et al. 2016; Albayrak et al. 2018; Eicher et al. 2018), autograders (Wang et al. 2018; Kyrilov 2014), and systems that passively monitor and then direct student progress (Paaßen et al. 2018) use AI, machine learning (ML), and deep learning technologies to store and process data and then communicate it to students and instructors. This exploitation of AI in education requires substantial funding and time for research, implementation, and assessment for the education community to understand the efficacy of the technology and its role in student persistence and subsequent on-the-job performance or success in graduate studies (Marr 2018; Polachowska 2019).

**Artificial intelligence** is the study of how to make computers perform tasks that appear to require intelligence when performed by humans. Machine learning and deep learning fall under this broad definition of artificial intelligence. **Machine learning** focuses on parsing and analyzing data in an automated fashion, without human intervention, to learn models for decision-making. Machine learning is considered a data mining technique. An algorithm that clusters data according to its similarities and differences is an example of machine learning. **Deep learning** is a subset of machine learning that relies on networks that mimic the way the human brain processes data and creates patterns to acquire decision-making ability.

A **chatbot** is a software program that converses with a human user. Chatbot ability ranges from those that conduct a shallow dialogue over a broad range of topics to those with deep knowledge and conversational ability over a well-scoped domain of discourse. The best are difficult to distinguish from human conversants. **Autograders** are software programs used to evaluate work produced by students with little or no human intervention. They can perform tasks ranging from scoring multiple choice tests to analyzing and grading essays. The findings produced by autograders range from binary (correct/incorrect) to conceptual feedback. Automatic review of essays is often combined with human review of the essays, with subsequent closer human examination of an essay if the automated and initial human results disagree. **Passive monitoring and guidance** can be integrated into an online learning system to compare a student’s activities to expected behavior. An instructor might learn through the system that several students engaged in online activities appear to be making similar errors, or when a particular individual appears to be lagging behind. They can also suggest interventions to the instructor, tailored to the difficulty encountered. Likewise, these systems can provide students hints about what they might try or modules to review, as well as feedback regarding how the student is progressing relative to the rest of the class.

Applications of AI-based education technology support learning in four ways: through automated tutoring, personalizing learning, assessing student knowledge, and automating tasks normally performed by the instructor (Lu and Harris 2018). Intelligent tutoring systems (ITS) produce statistically significant improvements in student learning outcomes, such as mastery and retention, when compared to traditional classroom teaching, independent textbook use, and non-AI computer-based instruction (Ma et al. 2014). However, experts point out that ITS curricula are rather inflexible due to technical challenges in accommodating user feedback, modified core standards, or content changes.

In addition to supporting improved student learning outcomes, the use of AI and ML in education has the potential to lead to improved teacher satisfaction (VanLehn et al. 2019a, b; Dietrich 2015). AI coupled with ML can provide 24/7 student support. It supports tracking student performance and aggregating student concerns. It can facilitate personalizing and adapting learning materials to individual students. These automated tools enable timely and passive assessment and more finely grained tracking of student knowledge and skill gains (Aleven et al. 2010; Arroyo et al. 2014). This assistance empowers the instructor, who can feel more confident in student opportunity to succeed, knowing that the students are receiving needed support that the instructor might otherwise struggle to provide. The instructor is able to devote time to creative activities and feedback beyond what the automated systems can provide, such as affective feedback and support (Wu et al. 2016; Duo and Song 2012).

The implementation of an online learning system requires a sophisticated digital ecosystem that incorporates the complex interactions among students, instructors, and content. It must include a sophisticated human-computer interface that supports access, monitoring, feedback, and assessment (Reyna 2011; Rezaei and Montazer 2016). The system can be built upon an existing e-business solution or learning management system, or arise from an array of independent modules. These systems are often cloud-based, providing services over the Internet, to provide maximum accessibility.

## Use of AI and ML in 2026

### Content Presentation

#### Opportunities

1. **Multifaceted presentation**, such as mobile computing, the Internet, natural language interfaces, gesture-based interfaces (Audinot et al. 2018; Bowman et al. 2008; Case 2018), silent speech recognition (Waltz 2019), and other advances in tools for user interface development have led to a richness in the modes of communication between humans and machines. Guided learning (Chi and Barnes 2014; Price et al. 2016; Ontañón et al. 2017) automates feedback tailored to student needs and gives students control over their learning (Zhou et al. 2016; Harackiewicz et al. 1987). Brownfield programming (Baley and Belcham 2010; Vujičić et al. 2018) allows students to learn programming by studying legacy systems. Simulation-based learning (Lateef 2010; *Official Site | Second Life—Virtual Worlds, Virtual Reality, VR, Avatars, Free 3D Chat* 2019; *OpenSimulator* 2019; OPNET Optimum Network Performance 2020) offers alternatives to real-world experiences. Content can be presented through multiple modalities, such as text, video, audio, or simulation. These advancements are extending our ability to interface with machines and each other. However, more work is needed for such systems to become highly reliable, robust, and widely available (Case 2018).
2. **Simulations** that embed AI and ML through interactive, realistic recreations of real-world scenarios provide additional means for presenting content. These allow students to gain experience with environments that would otherwise be inaccessible to them. Examples include high-performance computing and networking, in which access to commercially available or real-world systems would be costly or pose privacy or security concerns. Simulators, such as the OPNET network simulator (OPNET Optimum Network Performance 2020), simulate the behavior and performance of any type of network. To have a broad impact, a simulator must be easily ported to new environments or shared with low cost.
3. Content is also provided when students receive **automated feedback** on their work in progress. This includes automated grading of and feedback on assignments as student complete exercises (Kyrilov 2014; Wang et al. 2018), chatbots that respond to student questions (Eicher et al. 2018), or automated delivery of hints that guide a student’s next step in solving a problem (Paaßen et al. 2018). Grading rubrics based on a case-based reasoning paradigm reusing feedback on previous similarly graded coursework is an additional means of supplying content (Wiratunga et al. 2011).

#### Challenges

1. **Content management** is a challenging problem yet to be mastered. The more data that is accumulated, the greater the level of curation that is required. Intelligent storage and retrieval are needed to enable the presentation of context-relevant content (Miller 2017). This problem is being tackled by means such as observing the performance of a student over time and adjusting the type and frequency of automated hints provided to students by a behind-the-scenes passive assistant (Mostafavi and Barnes 2017; Peddycord-Liu et al. 2016). Each modality of presentation requires a different level of expertise for creation, delivery, and curation. Accessibility is also a challenge, raising concerns regarding how to present the same content in different modalities while accounting for various student (dis)abilities. Assignments and other activities clearly linked to learning objectives and tied to curricular requirements need to be both available and curated (Akbar 2013). Professional development on simulators and other tools and supporting documentation need to be available, as well.
2. **Feedback** provided via autograders ranges from binary responses indicating correct or incorrect to conceptual feedback. Detailed feedback enhances the learning experience (Kyrilov and Noelle 2016). There is evidence that instant binary feedback increases the likelihood that students will cheat on assignments (Kyrilov and Noelle 2015). Conceptual feedback can be provided through case-based reasoning. A case-based reasoner can analyze student errors, compare them to previously recognized errors, and retrieve and tailor specific guidance that proved useful to other students (Wiratunga et al. 2011; Kyrilov 2014).

#### Implementation Strategies

Content delivery can be cloud-based, server-based, or a combination of both. Fuad, Akbar, and Zubov present Dysgu (Fuad et al. 2018a, b) as an example of a cloud-based interactive learning environment. Dysgu personalizes and adapts out-of-class activities to satisfy individual student needs. Dysgu employs mobile technology to present activities that are smaller than traditional out-of-class activities. It incorporates social networking, which supports anonymous interaction and allows students to gauge their progress relative to the progress of other students.

In contrast, Isomöttönen, Lakanen, and Lappalainen’s TIM (The Interactive Material) is a document-focused system (Isomöttönen et al. 2019). TIM’s document-oriented user interface supports creating and editing learning materials, rather than managing course content. Instructors are able to track document sections (un)read by individual students. It also supports automated assessment, gamified learning through performance monitoring and display, and comprehensive tracking of submissions and user interactions with the system.

#### Research Questions

The appropriate use of online learning systems, their curation, and assessment are open areas. Several concerns need to be addressed on the path to full and efficient integration with learning experiences:

1. **Maintenance**. How will content be curated over time?
2. **Ownership**. Who will curate content over time?
3. **Relevance**. What is an effective mapping of tools to learning situations?
4. **Effectiveness**. Which data most accurately reflects the effectiveness of intelligent content delivery beyond measuring content knowledge?
5. **Affect**. Are affective measures more important? Do they reflect persistence, retention, or later mastery?
6. **Customization**. What functionality is needed to make assignment creation easier, less time-consuming, and flexible, allowing instructors to customize material to fit student level, knowledge, culture, and institutional requirements?
7. **Detail**. What level of detail and what type of information do students need to maximize the learning experience?

### Interactions and Communications

- **Classroom**
- Five minutes before the first class, Dr. Gomez logs into room system to turn on the affective computing machine. The cameras in the classroom quietly swivel toward the students’ seats and begin populating data to the instructor screen regarding individuals’ moods. Taylor Speek, the 27-year-old teaching assistant, starts meticulously drafting learning plans for students that are showing signs of poor engagement and difficulty grasping knowledge during the first session, making decisions based on an analysis of previous courses related to fluid dynamics. This class is particularly rich in information because all the students have signed waiver forms to be recorded and have their physiological and physical traits captured to monitor and notice patterns and trends in their progress. Looking at the pre-course assessment, Taylor notices that 83% of the learners showed signs of stress according to the data on their wearables, particularly in the area on Bernoulli’s principle. Taylor adds demystifying Bernoulli’s principle to the class agenda and gives Prof. Gomez a heads up.
- During the class, Alex runs into more confusion about Bernoulli’s principle and makes mouth movements so that her silent speech recognition device picks up the question without disrupting the class. “It makes you feel less self-conscious,” argues Alex. “My older sibling went into college thinking they would also pursue chemical engineering but then kept failing classes because they couldn’t get help and felt too embarrassed to ask.” The question is funneled to the class chatbot, which provides helpful resources curated by Prof. Gomez. The affective computing software on Alex’s computer registers that although the resources alleviate some of the stress, this student will need more support after class. The same question which has been asked in similar ways throughout the class is anonymously posted as a single topic to the class community for discussion afterward. Prof. Gomez proceeds with the final activity seamlessly, knowing that the peer learning forum used will foster more opportunities for students to reinforce what they learned from each other today. The chatbot reports back to the mainframe and automatically schedules a session between Taylor and Alex when they are both available.

#### Opportunities

**Fig. 1:**
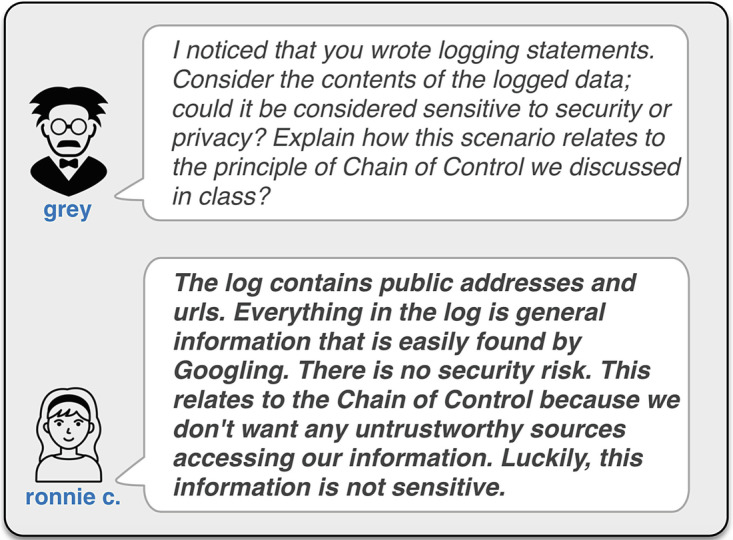
An example of Chen, Ciborowska, and Damevski’s automated reflection-in-action system. A student (ronnie c.) developing a secure mobile app is encouraged by the recommender system (grey) to learn about the security principle of chain of control (Reproduced from Chen et al. 2019)

1. **Interactions and communications** in an intelligent learning environment span user-initiated information retrieval and question answering, facilitation of informal or directed peer-to-peer communication, and student-facilitator interactions for guided learning. The facilitation of natural language interactions with systems, from storytelling to question answering, has been studied since the birth of artificial intelligence in the middle of the twentieth century (Winston 2016). Theories were devised for inferring the appropriate response to a question (Lehnert 1977; Wilensky 1977), and systems were constructed to allow novice users to ask about complex systems, such as Wilensky’s UNIX Consultant (Wilensky et al. 2000). Later systems supported natural language interfaces to databases (Androutsopoulos et al. 1995) and ultimately IBM’s Watson suite, designed for building conversational interfaces into any application, device, or channel (IBM 2018). Despite these advances, these interfaces lack deep knowledge and instead rely on massive amounts of data to train the system. While this is useful in most situations, being able to handle novel queries or mundane queries posed in an unusual fashion remains a challenge.
2. **Informal communication and collaborative problem-solving** are essential components of any learning experience. Students need to be able to develop a sense of community with their peers, as well as have access to expert knowledge and guidance from the instructor and teaching assistants. Dashboards allow the instructor to monitor individual student activity, present aggregate feedback on student activity, inform the instructor when a student appears to be having difficulty, and offer suggestions on additional approaches to presenting material that has the potential to enrich the learning environment.
3. Informal learning and co-curricular activities can be enhanced with **guided linear learning** tailored to a specific student’s needs and learning objectives. Fuad et al. (2018b) and Akbar (2013) use gamification to this end in their project “Active Learning for Out-of-class Activities to Improve Student Success.” In addition, points of intervention can be automatically identified, and the system can respond in collaboration with the instructor. Chen et al. (2019) develop this instructional strategy in their system that automatically delivers prompts to students based on the comments submitted when they commit software code to a source code repository. Their reflection-in-place app shown in Fig. 1 builds on a recommender system and guides students to reflect on their work in a meaningful way.

#### Challenges

Affect is an important component in human-human communication and can significantly influence learning experiences (Wu et al. 2016). Affective computing has three components: detecting the emotions of the user, expressing what a human would perceive as an emotion, and actually experiencing an emotion (Picard 2003).

- (1) and (2) Accurately **detecting** and **conveying** appropriate emotions is a complex task that is not well addressed in current intelligent systems.
- (3) The **integration** of emotions, mood, motivations, and personality contributes to user engagement (Fatahi and Moradian 2018). The lack of these dimensions is thought to lessen learner satisfaction with e-learning systems and lead to a higher drop rate of online courses. The challenges lie in the sophistication of the systems, the complexities of intercultural communication, and developers’ awareness of affect and ethics (Cowie 2014).

#### Research Questions

1. **Adaptation**. What is the appropriate mechanism for adaptation?
2. **Assistance**. What is the appropriate level of assistance to give students at various stages of learning?
3. **Dynamic adjustment**. Does the instructor have the ability to dynamically increase or decrease assistance?
4. **Automatic adjustment**. Can the level of assistance be automatically adjusted based upon a student’s performance?
5. **Affect**. What is the impact of multimodal emotion recognition and corresponding emotional response in e-learning systems?
6. **Ethics**. What is the role of ethics in human-computer communications and affective computing? What is an effective strategy for preparing the next generation of developers and educators to incorporate access and ethics into product design and content delivery?

### Learner Activities

- **Intervention**
- During Alex’s support session, Taylor comments on an interesting tidbit highlighted by the data dashboard. “Alex, it looks like when you took Process Control, the only aspect of the subject where you struggled was momentum balances, which relates to Bernoulli’s principle. Do you think that might be why you’re struggling?” Alex’s face lights up with this revelation and head nodding ensues. “Let me assign this additional activity you can do during your free time. I think it’ll help a lot with coming back to basics and slowly ramping back up. I think you should try and do this before the next session. It scaffolds the level of difficulty as you go and should challenge you in the correct areas. This app will customize the content to be more video-based since I know that’s what you seem to prefer. Is that correct? And don’t worry, a lot of students struggle with Bernoulli’s principle.” Taylor spends the remaining 15 minutes they have together providing emotional support, answering questions, adding points of clarification, and ensuring that Alex receives the additional materials. As the student leaves the room, another one takes his place, and Taylor’s dashboard changes to the appropriate student. When meeting with her master instructor, Taylor reflects, “These tools have made my work so much more meaningful and efficient. I get to support more students than I did in the past and really target the key areas of need.”

In the past decades, student activities and course assignments have evolved in new directions, from traditional take-home work to flipped classrooms (*Flipping the Classroom* 2019), from one-size-fits-all curriculum to personalized learning, and from paper and pen to digitized submissions requiring broadband Internet and access to personal devices. With these advancements, institutions have adapted their curriculum to more finely deliver and assess learning experiences more focused on personalization than in the past—creating an environment that is ripe for leveraging machine learning models. As such, the lines between activities and assessments have also blurred over time. In some cases, instructors become facilitators of experiences as opposed to disseminators of information. In this section, we explore the current opportunities, challenges, implementation strategies, and further research questions through the lens of uncovering best practices in intelligent instructional design.

#### Opportunities

1. **Interactive** and adaptive game-based learning is an increasingly popular area of investment for researchers focused on designing new digitized and innovative learning activities, which are often examples of constructivism in education. Constructivism explains that humans acquire and gain knowledge through experiences (Wadsworth 1996). LewiSpace by Ghali et al. (2016) is an example of an exploratory educational game that was developed with Unity 4.5 to teach a college-level chemistry lesson on drawing Lewis diagrams, which are structural representations of molecules. LewiSpace captures learners’ physiological traits such as electroencephalography (the measurement of electrical activity in different parts of the brain and the recording of such activity as a visual trace), facial expression, and eye movements throughout the game. LewiSpace also pulls data from a personality traits questionnaire in order to determine a learner’s performance and potential need for help. Experimenting with multiple machine learning algorithms, Ghali et al. (2016) found the highest level of accuracy in predicting failure rates using a logistic regression model.^1^ Future versions of LewiSpace will incorporate real-time measurements to adapt the experience to learner’s needs.
  Similar to Dysgu (Fuad et al. 2018b), an interactive mobile game that is currently manually scaffolded by instructors, and Epplets (Kumar 2018), an interactive tool for solving Parsons puzzles (Kumar 2017), LewiSpace presents a typical case study of the potential for simulations to be designed in more adaptive manners for the enhancement of constructivist learning. To support such efforts, Li et al. (2010) developed an adaptive course generation framework, which extracts course materials and learner profiles to help instructors design courses that are more advanced in handling multiple learning characteristics such as style and preference. This tool helps facilitate the alignment of course curriculum with the creation of adaptive activities.
2. **Supportive** elements in learning activities are crucial to ensure a relatable experience. Particularly in problem-based learning, enhancing critical thinking skills outside of the classroom may take the form of discussion-based activities. In such instances, intelligent learning tools such as MALESAbrain (Chiang and Fung 2004) encourage learners to judge their peers’ solutions before exploring further content. This information is used to rank and arrange learning issues in an effort to transform obligatory forums and chat rooms into rich discussion opportunities. This provides an opportunity to improve the quality of conversations inside and outside of the classroom, which may increase the opportunities for peer-to-peer learning.

#### Challenges

There are many remaining challenges toward leveraging artificial intelligence in regard to learner activities. Important challenges include:

1. Physiological traits such as facial expressions may be promising avenues for understanding and measuring engagement, but **interactive** and adaptive simulations experience obstacles when using metrics such as pupil dilation and emotions for predicting learner success (Ghali et al. 2016) due to the stimulating nature of the experience. These indicators may often add more noise in the form of irrelevant information or randomness in a dataset used by machine learning models trying to extract and determine specific causality, though future developments may overcome this sensory obstacle.
2. **Access** to personal devices that can successfully accommodate technological needs remains one of the most challenging obstacles of deploying learner activities. The digital divide persists for students within certain educational institutions and communities (Digital Divide Compounds U.S. Education Equity Problem, First-of-Its-Kind Survey Reveals 2018). As learner activities become increasingly demanding on devices and connectivity, ensuring that all students are equipped with the necessary means to access content will require careful attention. In order for engagement in learning to exit the classroom, simply equipping institutions will not suffice.

#### Implementation Strategies

Throughout the US education system, learner activities are often disseminated as instructors see fit to fulfill curriculum requirements and learning objectives. As such, instructors are a crucial part of the process for successfully deploying, tracking, and triaging these activities. As learning activities become more adaptive and personalized than instructors themselves, human instructors often become facilitators of experiences as opposed to disseminators of information.

Epplets (Kumar 2018) are a examples of software assistants designed to help students working alone learn good programming principles and algorithm design. The instructor retains the role of passing along knowledge to learners before the activity has begun. This small-scale integration of intelligence is an approachable first step in observing the outcome of allowing students to practice and assess at their own pace and level of rigor. Epplets enables a teacher to maintain a relatively hands-on approach in monitoring progress and providing aid where necessary. In more immersive experiences such as LewiSpace (Ghali et al. 2016), which replace an entire lesson, including the process of disseminating knowledge, instructors should be prepared to smooth the transition between classroom and simulation. In-person class time should be focused on deconstructing the virtual interaction, facilitating discussion, and processing learnings into applicable knowledge outside of the experience.

Instructors within institutions should provide a fluid and seamless experience inside and outside of the classroom for learners, particularly for flipped experiences.

#### Research Questions

There are a number of opportunities for further study. A few key examples include:

1. **Immersion**. How might we make learning experiences more immersive while maintaining transferrable real-world skills?
2. **Decision-Making**. How might instructors remain key decision-makers in adaptive learning experiences that often adjust with only learner data inputs?
3. **Indicators**. What might be the best measurements of competency and engagement for simulation-based activities?
4. **Noise**. Which psychological traits and data sources are the best indicators of learning and engagement?

### Assessment

- **Assessment**
- At the end of the semester, Prof. Gomez wraps up the last class with an essay-based exam. Students are asked to answer questions based on their greatest opportunities for improvement. Alex receives three prompts on inviscid flow and one on Bernoulli’s principle. The machine learning model is fine-tuned to Prof. Gomez’s competency-focused goal of measuring improvement to ensure that the students are well-rounded and confident in their abilities. “Why test a student on a topic that I know they’re an A+ on?” shares Prof. Gomez, “Let’s get straight to the point, what I care about is whether all of their skills are up to par and that we’re being effective in our delivery of learning. Throughout the course, we should have caught all of the pain points and now we’re just confirming.” As soon as Alex clicks “Submit,” the essays are automatically graded with a natural language processing tool. Prof. Gomez and Taylor also receive a new report on their dashboard, indicating that this final piece of data from the course has increased Alex’s likelihood of successfully graduating in chemical engineering.

A crucial component of determining the effectiveness of any learning program, intervention, and/or activity is being able to meet or exceed anticipated student learning outcomes. In optimal situations, all students would be equipped to successfully accomplish a variety of learning goals within and outside of traditional learning environments. However, it is common knowledge among students, educators, and parents that this is simply not the case. Leveraging artificial intelligence and, more specifically, machine learning methods can illuminate opportunities to quickly assess student performance, provide accurate feedback, proactively engage with students to an extent not possible without the intelligent intervention, and predict likely student outcomes. In the succeeding paragraphs, we explore the existing literature and opportunities within four major categories: (1) predicting performance, (2) reading and writing tasks, (3) zone of proximal development, and (4) personalized learning.

#### Opportunities

1. **Predicting Performance** is an essential area of study for machine learning applications in education. The traditional approach to monitoring student performance is to make assessment scores central to determining student achievement (National Research Council, Division of Behavioral and Social Sciences and Education, Center for Education, Board on Testing and Assessment, and Committee on the Foundations of Assessment 2001). To date, using these assessments to determine learner outcomes has been challenging due to ineffective and inefficient testing. Ogor (2007) proposes a methodology with a 94% success rating for monitoring students’ performance and predicting graduation status by capturing continuous assessment and examination scores. Alternatively, Ciolacu et al. (2017) have an interesting and novel approach to estimate student performance at examination through analyses based on neural networks, support vector machines, decision trees, and clustering. In this work, the authors leverage a blended learning course and a complete virtual course to test their model’s prediction accuracy. Another performance-related application of machine learning is predicting teamwork effectiveness by extracting objective and quantitative team activity data (Petkovic et al. 2012).
2. **Reading and Writing Tasks** are ubiquitous activities that all students encounter in higher education. EdX (*EdX: About Us* 2013), a massive online open course (MOOC) provider, has created a machine-based automated essay scoring (AES) application to assess student work at scale (Balfour 2013). Martinez et al. (2013) describe an AES that utilizes support vector machines, software encompassing supervised learning models for data classification and regression analysis. An AES must calibrate for each writing assignment and grade-level. They have been shown to correlate more highly with human raters than human raters among themselves (Shermis et al. 2010). AES offers immediate feedback to students though it is limited by unique speech elements such as humor. Nehm et al. (2012) developed another method with a high success rate. They created a “summarization integrated development environment” program, which assesses written explanations in biology using natural language processing. Assessment of a student’s reading level in order to improve the teacher’s ability to support an individual student’s learning is another opportunity to leverage support vector machines (Petersen and Ostendorf 2009). Although this application may be more useful in primary education, these principles may still support higher education English-as-a-Second-Language (ESL) students in all fields.
3. Vygotsky’s **Zone of Proximal Development (ZPD)** is an important concept that refers to the difference between what a learner can accomplish with help and by themselves (Chaiklin 2003). Traditional models of tutoring, office hours, etc. are difficult to scale and generally triggered by low grades, which occur after a student has an unsuccessful learning experience. Ahadi et al. (2015) explore machine learning methods, which automatically identify students in need of assistance by observing constantly accumulated early data such as students’ progress on assignments and behaviors in lectures. In an interesting contrast, Beck et al. (2008) investigate through the Bayesian evaluation and assessment framework whether or not tutorial interventions actually help students improve their outcomes and develop long-term and translatable skills.
4. **Personalized Learning** is an increasingly opportunistic challenge as learning at scale becomes more prevalent. How might learners improve their outcomes when in-person and virtual class sizes grow? García et al. (2007) focused on detecting students’ learning styles by evaluating the precision of Bayesian networks. The authors’ model infers a student’s style by capturing aspects of human behavior while the student is working with the system. Instead of assessing human behavior, Blikstein (2011) was interested in predicting it in the context of open-ended environments when performing tasks such as computer programming. AdaLearn (Alian and Al-Akhras 2010) creates a profile of learner responses to use for recommending content to learners. RubricAce (Wiratunga et al. 2011) improves rubric-based feedback to students using a case-based paradigm.

Finally, affective computing provides a promising avenue for machine learning applications as learner engagement, knowledge retention, and many other components of learning are influenced by emotion. Wu et al. (2016) provide a review of current trends and challenges with affective computing in education and learning. The authors identify common data collection and machine learning methods used. They also highlight opportunities to leverage insights to intervene at appropriate moments to improve learner trajectories.

#### Challenges

There remain a number of outstanding challenges toward fully developing artificial intelligence within synchronous and asynchronous environments. Key challenges in the four sections outlined above include:

1. **Predicting performance** (Ogor 2007; Ciolacu et al. 2017) presents ethical challenges if learners are given opportunities and support based on their anticipated grades and test scores from machine learning algorithms. Measures of performance on tests and grades are limited definitions of student achievement and real-world outcomes in STEM fields (Spector 2017). This approach limits the way in which we define learner success and evaluate whole-person skills that produce effective STEM graduates such as resilience, grit, or lifelong learning (Strauss 2017).
2. **Reading and writing tasks** that leverage AI require machine learning models to be trained for each assessment deployed at each grade level (Balfour 2013; Shermis et al. 2010; Nehm et al. 2012). This can be time-consuming and potentially costly for institutions that may not have the technical resources to implement AES tools. For others who may seek to purchase out-of-the-box solutions, this can create a black box where the AI is not explainable (Knight 2017). Instructors and administrators will be unable to clearly understand how written assignments are being graded.
3. Identifying appropriate interventions to help students reach their **zone of proximal development** requires a multitude of inputs, which may often be missed even by instructors in traditional classrooms. Predictive models created for specific situations may not be applicable and transferrable to other contexts, which may have variability in many aspects, such as teaching approach, materials, or group of students (García et al. 2007). An important part of this challenge arises from the difficulty of recognizing and accounting for these alterations in order to adjust the models being used.
4. **Personalized learning** powered by affective computing provides an opportunity to tap into human characteristics through facial recognition and other physiological traits (Wu et al. 2016). Unfortunately, there are a limited number of emotions that can be accurately identified, and there are concerns regarding the applicability of these defined traits to all demographics (Do 2019).

Several important questions remain, especially in the area of societal considerations. Can an AI be programmed to accurately account for racial, cultural, and religious differences? Can this be accomplished without controversy or running the risk of inappropriate racial or other profiling? Are there basic, humanistic beliefs that can be integrated into these systems to ensure equitable treatment and respect for all? Finally, there is the issue of consent. What challenges does a mixed classroom of those who consented and those who did not create? Does such a mixed classroom affect outcomes on both sides?

#### Implementation Strategies

In many cases, AI for assessment and evaluation are embedded within standard synchronous and asynchronous activities, e.g., evaluating test scores or written work. Data mining techniques are leveraged with machine learning models to passively determine desired predictions. As universities invite more remote students into their programs, there is an increased amount of potential data to be gathered, tested, applied, and analyzed. In instances such as Dysgu (Fuad et al. 2018b), instructors have manually assigned learner activities (replacing the judgment piece of what may become AI in the future) in order to increase student engagement outside of the classroom. This experiment provides a low-fidelity first step for institutions to test whether or not implementing AI in certain areas would be impactful to a student’s learning outcomes.

Additionally, the partnership between AI and the instructor is a key relationship to balance. Fully applying AI in a classroom or within institutions will require clarity in terms of role definitions and leveraging each party’s strengths. For instance, an instructor may be more effective in providing an emotion-based intervention after a machine learning algorithm has identified a disengaged learner, rather than having the instructor initiating an emotion-based response without understanding the current affective state of the learner.

#### Research Questions

There are a multitude of opportunities for future study. A few crucial examples include:

1. **Bias**. How might we train machine learning models to avoid replicating in-classroom, instructor, and institutional biases toward assessing certain demographics?
2. **Accessibility**. How might we leverage AI assessments in-classrooms and outside of classrooms to enhance all students’ sense of self-efficacy in STEM fields?
3. **Interactions**. Where might AI evaluations be best served to measure and improve student outcomes?
4. **Workplace Skills**. How might we leverage AI to better train, assess, and prepare STEM learners to thrive in the global workforce?

### Co-curricular Activities

Learning is being transformed by intelligent systems (Schmelzer 2019). Co-curricular activities are those which relate to and support an academic course of studies. AI-driven co-curricular activities are an opportunity to support online learning, the various ways people learn, and the rates at which they learn. Machine learning-based development of student profiles and customization of training materials allow instructors to draw upon a single curriculum while modifying content and presentation for individual users. Online textbooks and their interactive interfaces further support tailoring of content and personalization of delivery and feedback. They facilitate providing students hints as they work through assignments and conceptual feedback and as exercises are automatically assessed.

A learner interacts with material in many ways external to the formal educational event. Thus, the student experience consists of a wide range of interactions that may consist of athletic, scholarship, social, and service dimensions. Providing students with access to a range of these dimensions is a critical link in developing the overall quality of the student experience. Workshop participants identified several forms of interaction. They called for the augmentation of tutoring with personal, conversational education assistants, often referred to as autonomous conversational agents (IBM 2018). These and other integrated intelligent autonomous education agents must respond effectively to a learner’s questions and provide assistance with learning or assignment tasks. Learning systems need to reinforce concepts in a personalized fashion with additional materials to reinforce the curriculum. Furthermore, they need to allow students to learn at their own pace, to satisfy their own goals. Co-curricular education involves many opportunities and challenges. Co-curricular activities are implemented in a variety of ways and result in several open research questions.

#### Opportunities

X-FILEs workshop participants identified several characteristics of future learning and future students. Their consensus was that the basic pedagogy will still be delivered with new technology, and predicted that the best pedagogical ideas will be more fully realized. The Internet facilitates delivery of content to large numbers of individuals both synchronously and asynchronously, and the scale of delivery is expected to change. Feedback will become more automated, and content and delivery will be tailored to instructional objectives and the individual learner’s interests and needs. As institutions are called to do more with limited budgets, the importance of virtual environments over brick-and-mortar settings will increase.

Participants were asked how the teaching and learning process might be different in the future. Four responses stood out:

- Students will need to be more responsible for keeping up with the class. As a result, self-motivation will become essential.
- Access to educationally valuable locations will be freed from temporal and spatial constraints.
- Students will become more accountable for conducive learning.
- Learning will become more active, less passive.

A classroom environment that is conducive to learning entails staging the physical space, creating a communal environment, maintaining a positive climate and culture, and, most critically, convincing the students to become cooperative, active learners (Lynch 2016).

#### Challenges

Several challenges must be addressed to implement widespread adoption of automated intelligent co-curricular activities. These can be categorized as seeing a need for the systems, as well as acceptance, availability, assessment, and robustness.

1. **Need** Smaller programs that pride themselves on small classes and instructor-led courses might not recognize the value of automated intelligent co-curricular activities, or be reluctant to adopt them for fear of tarnishing their image of instructor-led, student-focused environments. In the age of COVID-19-motivated online learning, instructors are often encountering student questions that normally would be answered by readily accessible teaching assistants. These instructors are beginning to understand the need for automated activities.
2. **Acceptance and Availability** Social acceptance of automated agents that support co-curricular activities can present a challenge among both faculty and students. Workshop participants suggested that awareness of the value to student learning and independence and the availability of co-curricular activities can be raised through campus wide initiatives, such as panels, research symposia, industry, and examples. To increase adoption, one workshop participant advocated for holding a competition for faculty and students for the purpose of identifying instances of AI in the local institution’s environment. A leaderboard was proposed for recording, for example, the most creative entry or promising ways to increase student motivation, with prizes offered for the best examples. Identifying individuals on campus with experience and expertise and experience with automated co-curricular systems is essential when they are first introduced. Staff in an Instructional Technology office can prove invaluable to the rollout of this technology.
3. **Assessment** The efficacy of the co-curricular activities needs to be assessed. To accomplish this, institutional data from the library or institutional research unit could be made available to the community to build algorithms and related applications to measure the effectiveness of the activities.
4. **Robustness** Our students represent many abilities, countries, nationalities, communities, and cultures in many time zones, on many schedules, and with varying degrees of Internet connectivity. To be effective and accepted by a wide audience, the challenges of this diverse audience and these diverse environments need to be addressed. Systems need to be compliant with accessibility standards (US EPA 2013). Developers need to be aware of unconscious biases unintentionally embedded in systems that can misunderstand or alienate users (Eicher et al. 2018). In addition, a robust system would be able to understand and respond and present information in a variety of natural languages and at varying levels of abstraction (August 2012).

#### Implementation Strategies

AI-based co-curricular activities range from games to tutors to immersive simulations. Gamification platforms such as Classcraft, Rezzly, Seppo, Youtopia, and Kahoot! offer external motivation, such as rewards and leaderboards, and internal motivation, such as autonomy and mastery, to engage students in meaningful learning experiences (Goshevski et al. 2017). The instructor is able to provide or tailor content to increase relevance to the target content. AI chatbots, such as Jill Watson at Georgia Tech (Eicher et al. 2018), as well as Mtabe from Tanzania and LangBot in Ethiopia (Nsehe 2019), provide students personal tutors, tailored to instructional and learner needs.

Adaptive learning has the potential to promote access and quality at scale in higher education (Becker et al. 2018). Cavanagh et al. (2020) lay out the design of framework for adaptive learning and best practices for its use. The features of the design framework include objective-based learning knowledge units, personalized assessment and content, adaptive learning paths, alternate content, and procedurally generated questions. These systems can be implemented as standalone components or integrated into an existing learning management system. ALEKS (*Overview of ALEKS* 2020; Boyce and O’Halloran 2020) is one widely used for algebra. M-Shule from Kenya (Haba 2017) is an example of a data-driven personalized learning system for K-12 education.

Virtual labs present another opportunity for co-curricular activities that involve immersive simulations. Labster (*The Complete Guide to Virtual Labs* 2020) and PraxiLab (*Virtual Science Labs at Your Fingertips* 2020) offer commercially available virtual labs for secondary and higher education, as well as other informal learning needs.

Immersive platforms such as SimCity^®^, Second Life^®^, and the *Unity* real-time development platform are additional opportunities for implementing co-curricular activities that allow 24/7 access to engaging activities that support informal learning (August et al. 2016); Winkelmann et al. 2017).

#### Research Questions

Workshop participants offered a number of open research questions related to intelligent autonomous education agents in co-curricular activities:

1. **Implications of AI**. What are the implications of AI across co-curricular areas for the implementation and use of ML?
2. **AI enhancements**. How can AI expand the efforts in co-curricular activities?
3. **Levels of formality**. How do formal concepts and contexts differ from informal concepts and contexts?
4. **Human vs. machine intelligence**. What does comparing human learning and human intelligence to machine learning and machine intelligence tell us about what it means to be human?

## Conclusions

Development of robust, engaging, effective digital systems for learning must engage all classes of stakeholders from conception through implementation and evaluation. Such systems need to be integrated into the learning environment and into the routines of the instructors and students. Discussants at the 2018 X-FILEs Workshop identified several concerns:

- What is the relationship between the use of AI and ML in augmented learning systems and concerns such as ethics, empathy, equity, collaboration, and positive social change? Is there an obligation to consider them in parallel with the development of intelligent systems?
- What are the metrics and observations that would provide the greatest insight into the impact of AI in learning systems? Are the required data immediately available, or do they require longitudinal studies?

Addressing the first concern requires broad studies of innovative learning environments over diverse demographic groups and a range of higher education institution types. These will become more feasible over time as interactive learning environments are more widely adopted. Addressing the second concern requires looking to the longer term beyond gains in content knowledge and examining the affective impact of these learning opportunities, as well as development of critical thinking skills and fostering independent learning.

Many other questions remain to be considered:

- What are best practices for rolling out comprehensive online learning systems to ensure successful integration and achievement of learning objectives?
- What is the role of an intelligent online learning system in primary school? Secondary school? Higher education?
- How is an intelligent online learning system best integrated into primary education? Secondary education? Higher education?
- What is the role of the instructor in each?
- How are student/teacher interactions best integrated?
- What concerns do/should people have regarding limits on screen time, especially for younger students (Marr 2018)?

A rollout of the Summit Learning Platform (2017), a Chan Zuckerberg Initiative, points to multiple areas for future study, including integration of student/instructor integration, appropriate limits on screen time, parent acceptance, access to vetted resources, and controlled access to non-vetted resources. Community experiences in a Kansas school district reflect the need for more thought on these points before successful integration and achievement of learning objectives can be achieved (Bowles 2019).

## Footnotes

1 In statistics, the logistic model is used to model the probability of a certain class or event existing such as pass/fail, win/lose, alive/dead, or healthy/sick.
